# Rare case of a chronic rhinocerebral mucormycosis

**DOI:** 10.1016/j.mmcr.2024.100648

**Published:** 2024-04-09

**Authors:** Marie Louise Aicher, Jeanne Bisch-Karatas, Alexander Maurer, Franca Wagner, Martin Wartenberg, Stefan Zimmerli

**Affiliations:** a Department of Neurology, Inselspital, Bern University Hospital, University of Bern, Bern, Switzerland; b Department of Infectious Diseases, Inselspital, Bern University Hospital, University of Bern, Bern, Switzerland; c Department of Diagnostic and Interventional Neuroradiology, Inselspital, Bern University Hospital, University of Bern, Switzerland; d Institute of Tissue Medicine and Pathology, University of Bern, Bern, Switzerland

**Keywords:** Chronic rhinocerebral mucormycosis, Rhino-orbital-cerebral mucormycosis, Cavernous sinus syndrome, *Rhizopus arrhizus*, Cranial nerve palsy parainfectious vasculitis

## Abstract

We describe a case of rhino-orbital-cerebral mucormycosis (ROCM) in a diabetic patient. She presented with cavernous sinus syndrome and ischemia of the optic nerve. Initially unremarkable findings in the nasal cavity and paranasal sinus delayed early diagnosis. Within two weeks, a follow-up MRI showing progressive orbital inflammation, thrombosis of the cavernous sinus and erosive destruction of the left middle nasal concha together with necrotic black tissue in the left nasal cavity and destruction of the maxillary sinus demonstrated in a consecutively performed ethmoidectomy, finally gave way to diagnosis. Definite diagnosis was established by histopathology and culture. Treatment consisted of a combination of liposomal Amphotericin B, partial surgical resection and improved diabetes control. Despite insufficient surgical treatment, the progression of the disease was remarkably slow – a typical hallmark of chronic ROCM.

With this case report we aim to underline the difficulties in establishing a prompt diagnosis of ROCM and to remind readers of its chronic form.

2012 Elsevier Ltd. All rights reserved.

## 1 Introduction

Mucormycosis is a still rare, but emerging, usually fulminant fungal infection manifested by a variety of different syndromes with a high associated lethality [[1], [2], [3]]. It is caused by molds belonging to the order Mucorales, saprophytes found in organic material such as soil, manure and vegetables. The most prevalent clinical presentation globally is rhino-orbital-cerebral mucormycosis (ROCM) [1,3].

A clustering of fulminant cases especially from India in critically ill, often diabetic, patients during or after a COVID19-infection recently brought ROCM into the focus of international attention [4]. The main reason was presumably that the severe viral infection and its treatment, consisting of prolonged high-dose courses of corticosteroids, created optimal host factors for invasive infections by Mucormycetes. Pathogenically, initial defense mechanisms against Mucormycetes rely mainly on phagocytosis, which can be impaired both quantitatively (e.g. in neutropenia) and qualitatively. Both, hyperglycemia and acidosis, can lead to a dysfunctional phagocytosis by impairing chemotaxis as well as killing capacity of phagocytic cells [5]. Likewise, corticosteroids disturb the ability of macrophages to prevent germination of the spores in murine models [6]. This explains why the most commonly observed underlying condition for ROCM is diabetes mellitus, mainly with, but also without, associated ketoacidosis [3,7]. Another important pathogenic aspect is that hyphal growth critically depends on the availability of free iron. Both acidosis and excessive glycosylation of transferrin impair the binding and sequestration of iron by transferrin. The resulting elevated levels of available iron in diabetic ketoacidosis and hyperglycemic states serve as crucial fungal growth factor [5,8,9]. Once spore germination has led to hyphal growth, chemotactically attracted neutrophils have to attach to the hyphae to damage the cell wall using their oxidative cytotoxic system [8]. Otherwise, the fungus spreads rapidly and destructively into adjacent tissue. A hallmark of mucormycosis is the extensive angioinvasion with resulting vasculitis, vessel occlusion and tissue necrosis [9]. The necrotic areas offer optimal conditions for fungal growth because of high levels of free iron, lack of access for components of the immune system, and poor penetration of antifungal drugs. Bearing in mind the pathogenesis makes evident why in most cases treatment with antifungals and reversal of the underlying predisposing factors alone is not sufficient to control infection; radical debridement of all infected, necrotic tissues is of utmost importance [5,10,11].

Cases like the following with stagnation of infection despite incomplete debridement are therefore highly surprising. Although rarer than the acute and fulminant courses of ROCM, chronic cases with a more indolent course developing over weeks to months and occasionally resulting in apparent cure have been reported (reviewed in Refs. [12,13]). As the prognosis of these chronic cases seems to differ dramatically from the acute forms of ROCM, the following case report aims to remind readers of their existence.

## 2 Case

We present the case of a 76-year-old woman who reported a two-day history of frontal headaches, painful movement of the left eye as well as ptosis to colleagues at the emergency department of a peripheral hospital (day 0). On clinical examination, they found paresis of the left oculomotor nerve and reduced left periorbital sensation. Her past medical history included poorly controlled diabetes mellitus (HbA1c at admission 9.1 %, but no diabetic ketoacidosis) first diagnosed approximately twenty years ago, chronic renal failure, arterial hypertension, and dyslipidemia. Ophthalmoscopy showed left-sided perivascular ischemia, discontinuation of arteries, vasculitis, and ischemic alterations of the retina and papilla. A PCR test of gluteal vesicles for HSV-2 was positive. On day 2 a cerebral MRI revealed optic neuritis with inflammation extending to the orbital apex and minimal edema of the eye muscles. Lumbar puncture revealed polynuclear pleocytosis and increased protein levels but PCR and culture of cerebrospinal fluid (CSF) were negative. After a short initial treatment with valacyclovir for four days, ceftriaxone for two days, and high-dose corticosteroid therapy (500 mg methylprednisolone daily) for two days, the patient was referred to the university hospital for further care.

**Fig. 1):**
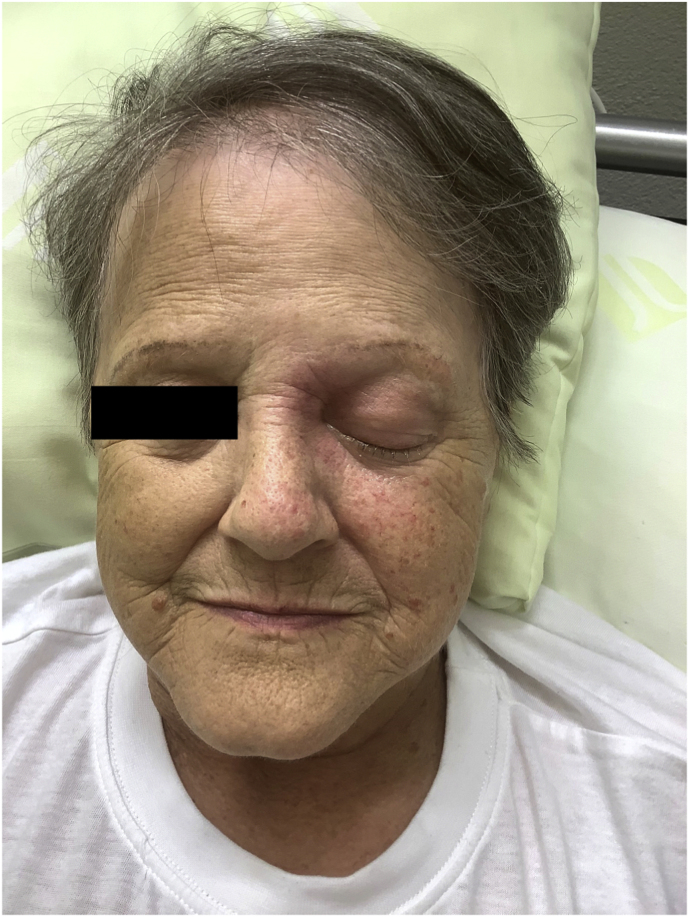
Clinical presentation of the patient with nasal and left sided periocular swelling, reddening and ptosis (day 8). The black bar covers her fully open right eye.

On examination on day 3, the patient presented with a left sided nasal and peri-ocular swelling and redness, ptosis and complete ophthalmoplegia, hypoesthesia in the trigeminal innervation area of V1 and V2 as well as a severe loss of vision (light/dark discrimination was initially preserved) (Fig. 1). Intravenous Co-amoxicillin was initiated for treatment of suspected bacterial periorbital cellulitis. Methylprednisolone was stopped and blood sugar levels were well controlled with a DPP4 inhibitor (Linagliptin) combined with rapid and long-acting insulin.

Laboratory analyses confirmed findings at the referring hospital demonstrating an elevated CRP (113 mg/L (normal range: <5 mg/L)), leukocytosis (16.2 x10^9^/L (normal range: 3-10.5 x10^9^/L)), mild mixed CSF pleocytosis (39/μL (normal range: 0–5/μL)), and a slightly increased CSF protein level (0.46 g/L (normal range: 0.2–0.4 g/L)). Again, no pathogen was detected (blood cultures, serology including galactomannan, and PCR for neurotropic infectious agents were all negative). CSF cytology showed a non-specific inflammatory reaction.

Another cerebral MRI on day 3 showed thrombosis of the left cavernous sinus as well as left sided periorbital cutaneous/subcutaneous changes consistent with cellulitis, in addition to the findings of the previous MRI. Rhinoscopy, performed five days after initial presentation to further investigate the cause of the cavernous sinus syndrome and the inflammation in the orbital and nasal regions, was unremarkable.

Within two weeks from symptom onset, the patient's visual acuity worsened, the field of vision was reduced in the right temporal field and she suffered from periorbital pain. Clinical examination now revealed a fixed left pupil. A repeat MRI on day 12 demonstrated progressive thrombosis of the cavernous sinus and inflammation of the left orbital cavity as well as new caliber irregularities and stenosis of the internal carotid artery compatible with a progressive infectious vasculitis. A CT scan on day 13 excluded relevant bone destruction of the craniofacial bones.

Due to the patient's clinical deterioration and the progression of the inflammatory process resulting in significant destruction of the nasal cavity, endoscopic ethmoidectomy was performed on day 13, which revealed black eschars of the mucosa suspicious of mucormycosis. Histopathology identified hyphae next to an extensive, florid, partly necrotizing inflammation involving blood vessels with occlusive vasculitis (Fig. 3). Later, tissue culture confirmed *Rhizopus arrhizus* (*R. oryzae*). Antifungal susceptibility testing revealed minimal inhibitory concentrations of 0.023 mg/L for amphotericin B, 0.75 mg/L for posaconazole, and 0.75 mg/l for isavuconazole.

On day 13, when the diagnosis of mucormycosis was first considered, the patient was started on high dose liposomal amphotericin B (10 mg/kg/d). In parallel, the left eye was exenterated and necrotic tissue was repeatedly debrided on days 15, 18 and 20. This mutilating intervention led to a distinct reduction of facial pain. Full removal of all infected tissue, however, was not achievable due to the involvement of the internal carotid artery. On day 28, amphotericin B was switched to oral posaconazole (300 mg/d after a loading dose of 600 mg on day 1). Therapeutic posaconazole steady state serum levels (>1.8 μg/ml [14]) were only achieved after 8 weeks when the daily dose was increased to 500 mg.

**Fig. 2a):**
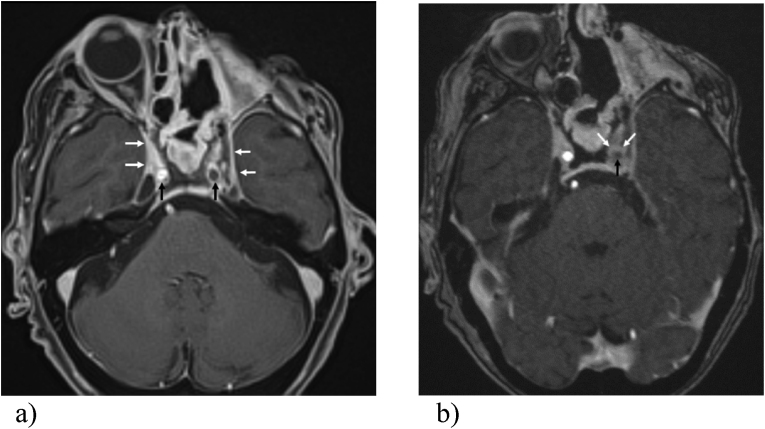
Follow-up MRI after left orbital exenteration. The axial T1-weighted starVIBE with fat saturation (slice thickness 1.5mm) after administration of intravenous contrast (Gadovist®) revealed progression of the inflammation on day 19 compared to day 3 and 6 with progressive bilateral involvement of the cavernous sinus (white arrows). A new filling defect of the left internal carotid artery is noted (black arrows), indicative of an occlusion of the vessel. The whole-brain post-contrast T1-weighted multiplane sequences showed perineural spread of the inflammation with extension along the course of the left mandibular nerve as well as contrast-enhancement of the right frontobasal pachymeninx. Fig. 2b)The 3D time-of -light MR angiography (slice thickness 0.6mm) after contrast confirmed the new occlusion of the left internal carotid artery with pathologically avid contrast enhancement of the thickened arterial wall (white arrows) and the missing flow void (black arrow). These MR images were performed on 3-T scanners (Siemens, Erlangen, Germany) 6 weeks after the initial diagnosis of the mucormycosis.

Surprisingly, the patient's condition stabilized despite incomplete surgical debridement. On day 32, she was discharged to outpatient care. Five weeks after the first symptoms (day 34), the patient was re-admitted because of epileptic seizures probably triggered by multiple small-volume strokes caused by a new, complete occlusion of the left internal carotid artery found on MRI. This finding was unchanged in the MRI on day 114, approximately 14 weeks after the start of treatment as depicted in Fig. 2a and b. The occlusion most likely resulted from (para-) infectious vasculitis of the left internal carotid artery, which also seemed to affect its branches as reflected by a decrease of diameters. After initiation and adjustment of an anticonvulsive treatment with levetiracetam there was no further evidence for recurrent seizures and the patient was discharged to a nursing home. In the outpatient setting, azole-therapy was switched from posaconazole to isavuconazole on day 57 because of laboratory-detected cholestasis. Therapeutic levels of isavuconazole (2.48 mg/L) were only achieved after 4 weeks. Five and a half months after the initial symptoms the patient died of urogenital *E. coli* sepsis most likely unrelated to her ROCM.

**Fig. 3):**
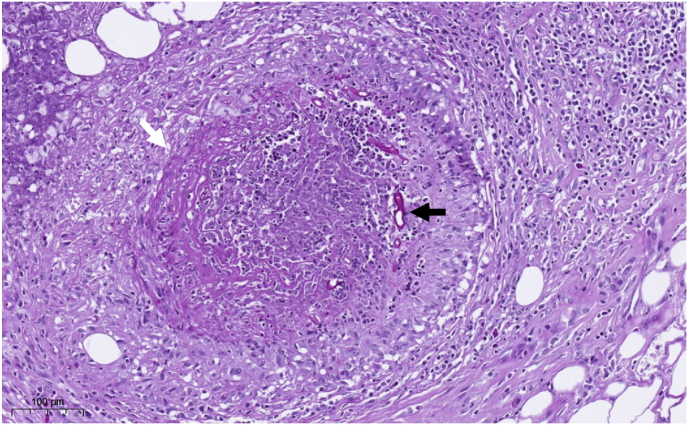
PAS stain 20x: Extensive florid, partly necrotizing inflammation involving blood vessels (white arrow) with occlusive vasculitis and fungal organisms (black arrow) near the optic nerve.

## 3 Discussion

ROCM is presumed to start with the germination of inhaled spores in the nasal cavity or the paranasal sinuses of a susceptible host. If untreated, infection usually spreads via the ethmoid or sphenoid sinus to the orbit and may further extend into neighboring tissues. Growth is destructive and angioinvasive [5,8]. Cavernous sinus thrombosis and internal carotid occlusion are associated with a very poor prognosis [15]. In the majority of published cases, ROCM is rapidly progressive. It develops over a few days and has a high associated mortality [15], unless treatment including radical surgery with complete removal of all infected and necrotic tissue, elimination or reversal of the underlying predisposing condition, and antifungal drug therapy is initiated promptly [5,10,11].

We present a case with a different, chronic course of this infection and a favorable evolution despite incomplete surgical treatment. To our knowledge, there are only a few case reports of similar chronic cases, reviewed 1996 [12] and 2016 [13]. Due to these differing courses of ROCM, several authors distinguish two clinical entities of rhino-cerebral mucormycosis, an acute versus a chronic form [12,13,16]. There is no exact definition of chronicity, but classically it is defined by duration of symptoms of at least four weeks. Case reviews report a median duration of 7 months [12,13] versus less than 2 weeks in most published cases of acute ROCM [11,15,17]. The reasons for these two different clinical presentations are not understood, yet. Although one might assume that the immune status of the host could be a distinguishing factor between the two forms, there is no clear evidence in the literature for this thesis. The infection seems to affect immunocompetent patients in approximately 10 % [17] of the acute and up to 26 % of the chronic form [13]. Correspondingly, in most of the published cases of chronic ROCM, patients had an underlying condition affecting the immune status, mainly poorly controlled diabetes mellitus with, but also without ketoacidosis like in our patient [12,13,16]. Likewise, the presenting features of our patient, which do not seem to differ significantly between acute and chronic forms, were consistent with described cases: in a review of 152 cases worldwide, the most common symptoms were periorbital swelling (27 %), fever (26 %), decreased vision (20 %), ptosis (18 %), ophthalmoplegia (15 %) and periorbital pain (14 %) [15]. Particularly, acute localized pain radiating to the eye like in our patient is one of the clinical features necessary for the diagnosis of a probable invasive sino-nasal mold infection in severely immunocompromised patients according to the consensus definitions of the Infectious Diseases Group of the European Organization for Research and Treatment of Cancer and the Mycoses Study Group [18]. Despite retrospectively compatible symptoms and risk factors in our patient, it took two weeks to make the correct diagnostic. One explanation might be the rare occurrence of ROCM and its lack of specific clinical presentation, laboratory results or MRI signs. Moreover, the initial lack of evident pathological manifestations in the nasal cavity and paranasal sinuses in both, MRI and rhinoscopy were the most misleading factors. Interestingly, in their case reviews from 1996 resp. 2016 Harril et al. as well as Gutierez et al. also found a high incidence of occlusion of the cavernous sinus and/or the internal carotid artery in their cases of chronic ROCM [12,13], which is an indicator of a poor prognosis in the acute form [15].

Because of the initially predominant findings, we discussed several differential diagnoses causing periocular inflammation and cavernous sinus syndrome. Other infectious diseases such as bacterial (facial or orbital cellulitis, abscess, empyema, cerebral tuberculosis) or viral infections (zoster ophthalmicus), para-infectious phenomena (vasculitis due to infection with hepatitis B and C viruses or *Mycoplasma pneumonia*), malignant diseases such as lymphoma, and autoimmune diseases such as a temporal arteritis, systemic lupus erythematosus or granulomatosis with polyangiitis (GPA) were considered. Particularly, we discussed granulomatous diseases such as Tolosa Hunt syndrome or neurosarcoidosis. Initial suspicion of temporal arteritis even resulted in a short course of high-dose corticosteroids, which, in an acute fungal infection, could have resulted in further weakening of the immune response.

Finally, two findings gave way to the correct diagnosis and treatment: first, a follow-up MRI showed not only progressive orbital inflammation and thrombosis of the cavernous sinus but also erosive destruction of the left middle nasal concha. Second, a consecutively performed ethmoidectomy demonstrated necrotic black tissue (eschar) in the left nasal cavity as well as a destruction of the maxillary sinus. Definite diagnosis in our patient was established by histopathology and culture. Given that in most of the published cases of chronic ROCM, diagnosis was based on histopathology alone, it is not clear if there is a difference in causative species in chronic cases. Our case argues against this hypothesis, as *Rhizopus arrhizus* is the most frequently isolated species in ROCM in general [8].

The progression of the disease in our patient was unexpectedly slow despite incomplete surgical debridement and serum antifungal drug levels that were often below the recommended therapeutic range. A comprehensive removal of all infected tissue was impossible because of the involvement of the cavernous sinus and the carotid artery. The slow disease progress in our patient is in accordance with the published cases of chronic ROCM. The prognosis of this form seems to be more favorable: Harril et al. reported in 1996 a survival rate of 83 % for the chronic form, whereas Jeong et al. observed a mortality rate of 43 %–75 % in cases with orbital and/or cerebral involvement between 2000 and 2017 [2,12]. Some authors even considered that a less aggressive surgical treatment might be sufficient [12,16], as suggested by the case of our patient. Finally, only the slow course of the disease with survival over months despite incomplete debridement led us to classifying our patient's case as chronic ROCM, [12,13].

In summary, acute and chronic ROCM differ, according to current knowledge, only in the course of the disease itself. It is notable that comprehensive removal of all infected tissue seems not to be a prerequisite for successful treatment of chronic ROCM [12,16], whereas in acute forms remaining infected tissue will result in relentless spread of the fungal infection into adjacent tissue and eventually treatment failure. As of to date, data about the chronic variant of the disease are so sparse that it is essentially an unknown entity [11]. This report should remind readers of the existence of such cases. Furthermore, it exemplifies the difficulty of establishing a timely diagnosis in ROCM. An increasing incidence of invasive fungal infections in the future is likely due to the demographic change, the rising prevalence of diabetes mellitus, and the number of patients receiving immunosuppressive therapies. Therefore, deeper knowledge of the different entities of mucormycosis, some with a prognosis that is better than previously assumed, is warranted.

## CRediT authorship contribution statement

**Marie Louise Aicher:** Conceptualization, Writing – original draft, Writing – review & editing. **Jeanne Bisch-Karatas:** Conceptualization, Writing – original draft, Writing – review & editing. **Alexander Maurer:** Supervision, Writing – review & editing. **Franca Wagner:** Resources, Writing – review & editing. **Martin Wartenberg:** Resources, Writing – review & editing. **Stefan Zimmerli:** Conceptualization, Supervision, Writing – original draft, Writing – review & editing.
